# Identification of a novel link connecting indole‐3‐acetamide with abscisic acid biosynthesis and signaling

**DOI:** 10.1111/nph.70819

**Published:** 2025-12-08

**Authors:** José Moya‐Cuevas, Paloma Ortiz‐García, Adrián González Ortega‐Villaizán, Irene Viguera‐Leza, Andrés Pérez‐González, Javier Paz‐Ares, Carlos Alonso‐Blanco, Jesús Vicente‐Carbajosa, Stephan Pollmann

**Affiliations:** ^1^ Centro de Biotecnología y Genómica de Plantas Universidad Politécnica de Madrid (UPM)–Instituto Nacional de Investigación y Tecnología Agraria y Alimentación (INIA/CSIC) Campus de Montegancedo 28223 Pozuelo de Alarcón (Madrid) Spain; ^2^ Department of Plant Molecular Genetics Centro Nacional de Biotecnología (CNB‐CSIC) 28049 Madrid Spain; ^3^ Departamento de Biotecnología‐Biología Vegetal, Escuela Técnica Superior de Ingeniería Agronómica, Alimentaria y de Biosistemas Universidad Politécnica de Madrid (UPM) 28040 Madrid Spain

**Keywords:** abiotic stress response, natural variation, plant hormone crosstalk, reverse genetics, root growth inhibition

## Abstract

- Plants orchestrate their developmental processes and responses to environmental stimuli through a sophisticated network of small signaling molecules, termed phytohormones. Among these, auxins are recognized for their role in promoting plant growth. However, indole‐3‐acetamide (IAM), an auxin precursor, has been observed to inhibit primary root elongation. The molecular mechanism underlying this inhibitory effect remains largely unexplored.
- A comprehensive genome‐wide association study (GWAS) conducted on a highly diverse collection of 166 wild Arabidopsis accessions from the Iberian Peninsula has identified several genomic regions associated with reduced IAM sensitivity under controlled *in vitro* conditions. This study highlighted *ABA3* and *GA2ox2* as possible candidate genes.
- Molecular and structural analyses suggest that the inhibition of primary root elongation induced by IAM is intricately associated with the enhanced production of abscisic acid (ABA) involving ABA3.
- Studies employing mutant and reporter lines have confirmed that IAM activates ABA signaling, thereby revealing a novel interaction between the auxin precursor IAM and ABA and suggesting an independent role for IAM as a signaling molecule in plant hormone crosstalk.

## Introduction

For the implementation of intrinsic growth programs, as well as for the adaptation to their ever‐changing environments, plants utilize a limited number of signaling molecules, commonly referred to as plant hormones. These are defined as naturally occurring substances that function at submicromolar concentrations to drive physiological processes either locally or in distant tissues (Santner *et al*., 2009; Davies, 2010). Different groups of plant hormones independently control specific sets of biological functions. There is, however, mounting evidence supporting the widely accepted perspective that the overall effects of plant hormones are shaped by the complex interplay of the different phytohormones and their interactions within cells, achieved through the integration of their signaling pathways in both space and time (Wolters & Jürgens, 2009; Depuydt & Hardtke, 2011; Ortiz‐García *et al*., 2023; Wong *et al*., 2023; González Ortega‐Villaizán *et al*., 2024). Plant hormones influence the development of a responding tissue by inducing transcriptional reprogramming of the corresponding cells. Transcriptional alteration can, consequently, result in physiological and metabolic adjustments (Hsu *et al*., 2021; Yoshida & Fernie, 2023). These initiated responses are additionally dependent on the specific properties of the responding tissues regarding their sensitivity and responsiveness to the given interacting signaling compound classes (Bradford & Trewavas, 1994; Knight & Knight, 2001).

Among plant growth‐promoting phytohormones, indole‐3‐acetic acid (IAA), the most important endogenous auxin, plays a pivotal role in numerous plant growth and developmental processes, including cell division and expansion, meristem maintenance, differentiation, tropisms, apical dominance, senescence, leaf abscission, and flowering (Woodward & Bartel, 2005; Teale *et al*., 2006). Recent research has demonstrated the importance of stringent spatiotemporal regulation of auxin biosynthesis and its correlation with the aforementioned processes (Zhao, 2010; Xu *et al*., 2018). The primary precursor for IAA biosynthesis in plants is l‐tryptophan (l‐Trp). The indole‐3‐pyruvic acid (IPyA) pathway constitutes the principal route for IAA formation. This pathway proceeds from l‐Trp through IPyA to IAA and involves the tryptophan aminotransferases TRYPTOPHAN AMINOTRANSFERASE OF ARABIDOSIS 1 (TAA1) and TRYPTOPHAN AMINOTRANSFERASE RELATED 2 (TAR2). Several alternative pathways, including the tryptamine pathway, the indole‐3‐acetaldoxime pathway, and the indole‐3‐acetamide (IAM) pathway, are also considered to contribute to IAA biosynthesis under specific conditions (Pollmann *et al*., 2006; Kasahara, 2016).

The IAM pathway comprises two enzymes: a tryptophan 2‐monooxygenase (tms1, iaaM, aux1) that catalyzes the conversion of l‐Trp to IAM, and an IAM‐specific amidohydrolase (tms2, iaaH, aux2), which subsequently hydrolyzes IAM into IAA. Initially, this pathway was believed to occur exclusively in certain phytopathogenic bacteria of the genera *Agrobacterium*, *Pantoea*, and *Pseudomonas*. However, this understanding changed with the identification and characterization of the first IAM‐specific amidohydrolases of Arabidopsis (*At*AMI1), tobacco (*Nt*AMI1), and several other plant species (Pollmann *et al*., 2003; Nemoto *et al*., 2009; Sánchez‐Parra *et al*., 2014). More recent research has demonstrated that *ami1* T‐DNA insertion mutant seedlings exhibit hypersensitivity to osmotic stress, and it has been proposed that the transcriptional suppression of *AMI1* expression by abiotic stress signals serves as a driver of proper adaptation mechanisms to this type of stress (Moya‐Cuevas *et al*., 2021; Pérez‐Alonso *et al*., 2021). Previous research has indicated a possible regulatory relationship between IAM and the biosynthesis of ABA. However, natural variation has not been utilized as a methodological approach to investigate the role of IAM as a potential signaling molecule, distinct from IAA.

In this study, we present the findings of a genome‐wide association analysis aimed at identifying loci linked to the inhibitory effect of IAM on primary root growth, utilizing 166 Arabidopsis ecotypes from the Iberian Peninsula. Genetic analysis of a small number of potential candidate genes indicated that *ABA3* is involved in the IAM‐triggered negative regulation of primary root growth. Additionally, an investigation into the networks of IAM and ABA coregulated genes uncovered a putative core response module that plays a role in managing responses to water and osmotic stress in Arabidopsis.

## Materials and Methods

### Plant material

A previously published collection of 235 *Arabidopsis thaliana* (L.) Heynh. accessions from the Iberian Peninsula (Arteaga *et al*., 2021) was synchronously grown to multiply the seed material. After the plants completed their life cycle, the seeds were harvested and stored under homogeneous conditions (22°C, 50% relative humidity RH). For the genome‐wide association study (GWAS) experiment, accessions that showed low or null germination rates were discarded. A resulting subset of 166 accessions was selected for the final experiment. The metadata of all the employed accessions are provided in Supporting Information Table S1. Each accession corresponds to the self‐progeny of a randomly sampled individual plant per population.

In addition, the following Arabidopsis accessions and mutants, respectively, were used: Col‐0 (stock N1092), 6 × ABRE_A::erGFP and 6 × ABRE_R::erGFP (Wu *et al*., 2018), pMAPKKK18::Luc^+^ (García‐Maquilón *et al*., 2021), *aba3‐1* (Léon‐Kloosterziel *et al*., 1996), *abi5‐7* (Nambara *et al*., 2002), *ga2ox2–1* (Rieu *et al*., 2008), and *ga2ox q* nls GPS1 (Rizza *et al*., 2021).

### Root growth inhibition assay

Seeds were surface‐sterilized, using sodium hypochlorite and 70% (v/v) ethanol, and then stratified at 4°C for 2 d. In this study, 15–20 seeds per accession were initially grown in six‐well plates, containing 10 ml per well of liquid 0.5× Murashige and Skoog (MS) media (Murashige & Skoog, 1962) supplemented with 1% (w/v) sucrose, and germinated for 4 d under controlled long‐day growth conditions (22°C, 16 h light : 8 h dark). Thereafter, the liquid media was replaced by 0.5× MS media containing only 0.1% (w/v) sucrose. Half of the seedlings were treated with 10 μM IAM (from 1 mM stock in ethanol), while the other half (control replicates) was mock treated with the corresponding volume of IAM‐free ethanol. After 12 d, the seedlings were spread out on a solid support and photographs were taken to facilitate the measurement of the primary root length using the Fiji software (Schindelin *et al*., 2012). The primary root length of all tested accessions was normalized against the results obtained for Col‐0 reference plants that were added to each six‐well plate.

### Genome‐wide association study

The genome sequences and single nucleotide polymorphism (SNP) data for the 166 Arabidopsis accessions used for GWAS are included in the 1001 Genomes project (Genomes Consortium, 2016) and in the publicly available databases of the online applications for GWA Mapping in Arabidopsis, GWAPP (Seren *et al*., 2012), and easyGWAS (Grimm *et al*., 2016). These platforms were used to conduct the GWAS using as input the obtained data for the normalized relative primary root length reduction percentage in response to IAM treatment (PR length_Control_ – PR length_IAM_). A mixed linear model was performed to obtain candidate SNPs which were filtered both by minor allele frequency ≥0.05 and a suggestive relaxed *P*‐value criterion <10^−5^ to consider possible IAM response‐related genomic areas. Genes enclosed within a 10 kb region around the identified SNPs were listed and ranked.

### Modelling of the ABA3 protein structures

The 3D structures of the reference ABA3_Ref protein from Col‐0 and the altered ABA3_Alt version containing all the common SNPs in the Arabidopsis accessions that showed reduced sensitivity toward IAM were modeled by using a homology‐based approach. The 1.65 Å crystal structure of the cysteine desulfurase (SufS) from *Mycobacterium tuberculosis* (PDB: 8ODQ) (Elchennawi *et al*., 2023) and the 1.80 Å crystal structure of *Synechocystis* sp. PCC 6803 cysteine SufS [PDB: 1T3I] (Tirupati *et al*., 2004) deposited in the Research Collaboratory for Structural Bioinformatics (RCSB) Protein Data Base were used as reference structures. The different structural models were generated by using both the Phyre^2^ protein fold recognition server (Kelley *et al*., 2015) and the I‐TASSER protein structure prediction server (Yang & Zhang, 2015). The structural comparison of the obtained models for ABA3_Ref and ABA3_Mod was performed using PyMOL v.2.5.5 (https://pymol.org/) and the InterPro (https://www.ebi.ac.uk/interpro/) protein classification tool.

### Analysis of luciferase activity

For *in vivo* evaluation of changes in ABA signaling activity in response to treatment with IAM, bioluminescence measurements were performed using the pMAPKKK18::Luc^+^ reporter line. To this end, the reporter line was grown vertically for 10 d on 0.5× MS plates containing 1% (w/v) sucrose. The plants were then treated with a mock solution or a solution containing 20 μM IAM for 2 h, before monitoring luciferase activity using a cooled CCD camera (NightOwl II LB 983 NC‐100; Berthold Technologies, Bad Wildbad, Germany). To visualize the luciferase activity, plates were sprayed with 100 μM luciferin and imaged after an incubation time of 40 min.

### Confocal laser scanning microscopy

ABA signaling activities were also investigated in the roots of mock‐ and IAM‐treated seedlings of ABA signaling reporter lines 6 × ABRE_A::erGFP and 6 × ABRE_R::erGFP using a Leica SP8 microscope with the Leica Application Suite (Las AF Lite) X software. As described above, the reporter plants were incubated for 2 h in a mock solution or in a solution containing either 20 μM IAM or 10 μM ABA. Thereafter, the green fluorescent protein (GFP) was excited at 488 nm using an Argon multiline laser and detected using a 494–596 nm broadband filter.

### RNA isolation and gene expression analysis by quantitative reverse transcription polymerase chain reaction (qRT‐PCR)

For the extraction of total RNA, 100 mg of plant tissue from 10‐d‐old sterilely grown seedlings were harvested as previously described (Oñate‐Sánchez & Vicente‐Carbajosa, 2008). First‐strand synthesis was conducted using M‐MLV reverse transcriptase and oligo(dT)_15_ primer, following the instructions of the manufacturer (Promega, Madison, WI, USA). Two nanograms of cDNA were used as a template in each quantitative polymerase chain reaction (qPCR). cDNA amplification was performed using the FastStart SYBR Green Master solution (Roche Diagnostics, Barcelona, Spain) and a Lightcycler 480 real‐time PCR system (Roche Diagnostics), according to the supplier's instructions. The relative transcript quantification was calculated employing the comparative 2^−∆∆*C*T^ method (Livak & Schmittgen, 2001). As reference genes, we used *APT1* (At1g27450) and *GAPC2* (At1g13440) (Czechowski *et al*., 2005; Jost *et al*., 2007). All experiments were carried out using biological triplicates. In addition, three technical replicates per biological replicate were analyzed. See Table S2 for primer sequences.

### Comparative analysis of selected gene sets

The functional classification of DEGs was performed using the MapMan v.3.6.0R1 software (Thimm *et al*., 2004), paying special attention to DEGs related to plant hormones. Furthermore, functional relationships between the DEGs were investigated employing the stringApp v.2.0.3 (Doncheva *et al*., 2019), and EnrichmentMap v.3.3.6 (Merico *et al*., 2010) in Cytoscape v.3.10.1 (Shannon *et al*., 2003). To analyze the importance of the nodes in the inferred networks, the nodes with the highest degree of connectivity (k) and betweenness centrality (BC) were examined in closer detail.

### Statistical analysis

The statistical assessment of the data was performed using the JASP v.0.18.3 software (https://jasp‐stats.org/). Student's *t*‐test was employed to compare two means. Statistical differences of more than two means were determined by one‐way ANOVA and Tukey's *post hoc* test for pairwise comparisons. Results were considered significant when the *P*‐value <0.05.

## Results

### IAM treatments highlight multiple genomic regions and candidate genes

**Fig. 1:**
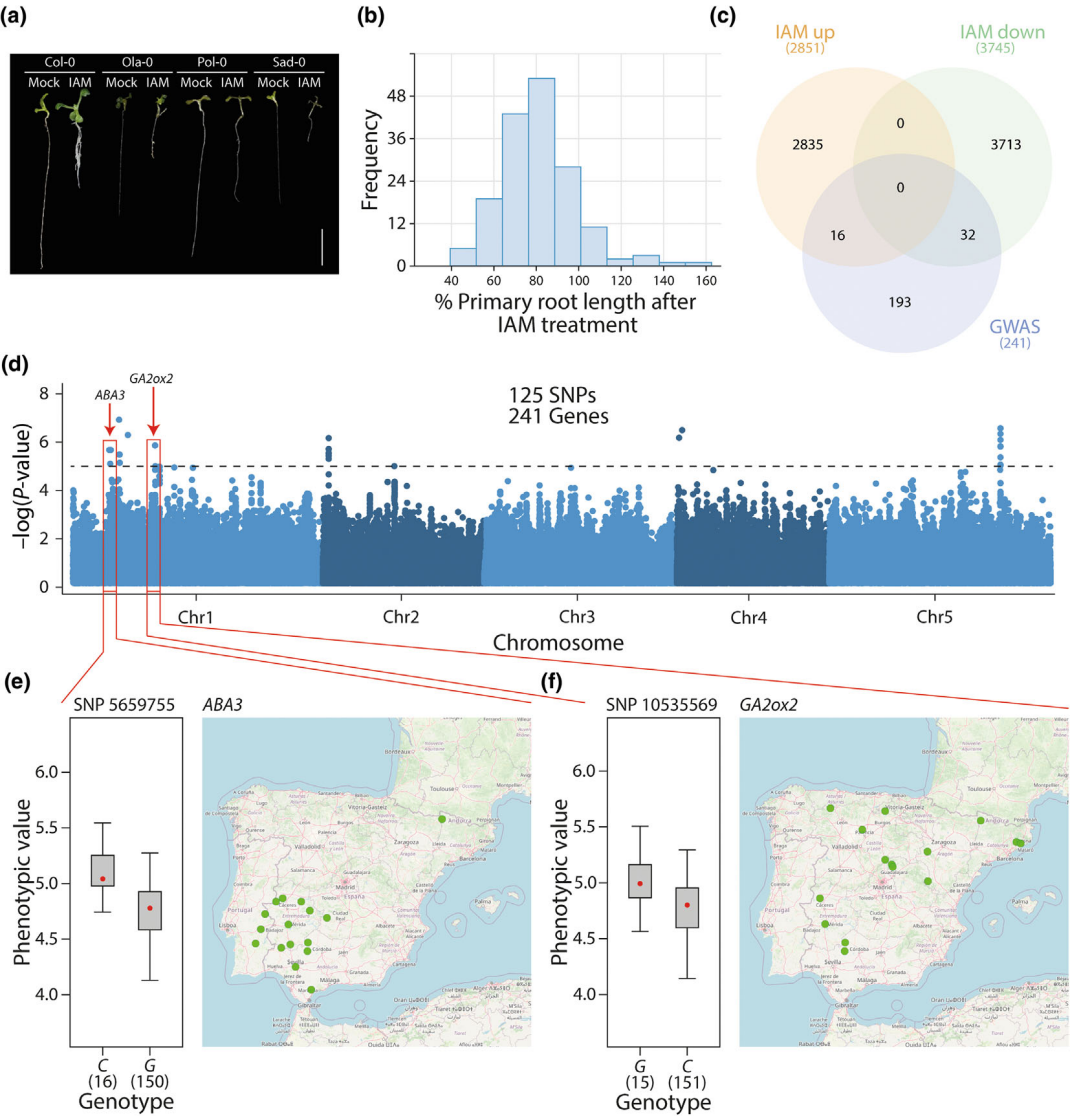
*Arabidopsis thaliana* accessions from the Iberian Peninsula display significant natural variations in their root growth response to indole‐3‐acetamide (IAM). (a) Representative images of four randomly selected accessions. (b) Frequency distribution of IAM‐triggered changes in primary root growth in 166 Arabidopsis accessions. (c) Venn diagram analysis of described IAM‐responsive genes and the candidate genes identified through the GWAS approach. (d) Manhattan plots depicting IAM‐induced primary root growth repression based on the linear mixed model. The dashed line represents −log_10_(*P*‐values) > 5. (e, f) Normalized phenotypic values and geographic distribution of accessions exhibiting the minor frequency allele of SNP 5659755 (*ABA3*) and SNP 10535569 (*GA2ox2*). The box plots illustrate the median (red dot), the 25–75% quartiles (gray box), and the extrema (whiskers) of the compared data.

To identify novel molecular components involved in IAM‐dependent repression of primary root growth, a set of 235 Arabidopsis accessions from the Iberian Peninsula was utilized to conduct a GWAS. Following synchronization of all accessions, 166 readily germinating natural accessions were selected for mock and IAM treatments and subsequent phenotypic analysis. As illustrated in Fig. 1, a substantial range of variability was observed among the accessions tested regarding their response to IAM in the growth medium. To standardize the responses across various accessions, each six‐well plate included Col‐0 (Columbia) seedlings as a reference for normalization throughout the experiments conducted. The subsequent Shapiro‐Wilks test confirmed the normal distribution of the data (*W* = 0.952, *P* = 2.21 × 10^−5^). Although IAM repressed root growth in the majority of accessions, a promotive effect was observed in 11% of the accessions (Fig. 1b). Normalized root response data obtained after mock and IAM treatment of the different accessions (Table S1) were utilized to perform the GWAS analysis using the easyGWAS application (http://easygwas.biochem.mpg.de). To identify potential genomic regions associated with the tested phenotype, a relaxed *P*‐value threshold (<10^−5^) was established (Fig. 1d). Candidate genes located within a 10 kb region around the associated SNPs were compiled (Table S3). The SNP with the highest score (−log_10_(*P*‐value) = 6.02) was SNP 16531216 on chromosome 5. Of the five genes located around the SNP, only two have been functionally characterized: *GLABRA 3* (At5g41315), which is related to trichome development (Bernhardt *et al*., 2003), and *UBIQUITIN CONJUGATING ENZYME 4* (At5g41340), which is involved in sugar metabolism and leaf senescence (Wang *et al*., 2022). For all SNPs that met the established criteria, a total of 241 possible target genes were obtained. Among the putative candidate genes, 22 transcription factor (TF) genes were identified, including three Lateral Organ Boundary genes and two Agamous‐like genes, as well as 12 development‐related genes, including the secondary cell wall‐related Arabidopsis NAM, ATAF1/2, and CUC2 (NAC) domain *NAC10*, and the *HAIRY MERISTEM 3* (*HAM3*) gene (Table S3). However, few of these genes exhibited documented expression in roots. The functional classification of the genes using the MapMan application (v.3.6.0R1) provided additional evidence for a limited number of candidate genes related to plant hormones.

From the candidates presented in Table 1, the genes *ABA3* (At1g16540) and *GA2ox2* (At1g30040) were selected for further analysis due to their presence among the 16 and 31 GWAS candidate genes that have previously been reported to exhibit increased and decreased expression (Fig. 1c), respectively, in response to a short‐term IAM treatment (Ortiz‐García *et al*., 2022).

**Table 1:** Functional classification of plant hormone‐related Arabidopsis genes detected by the genome‐wide association study (GWAS) analysis using MapMan.

MapMan Bin code	AGI	Description	Significance threshold
17.2.3	At1g16510	SAUR41 | auxin‐responsive family protein	1.64E−05
17.3.3	At3g61460	BRH1 | Brassinosteroid‐responsive RING‐H2	6.23E−05
17.6.1.11	At5g51310	Gibberellin 20‐oxidase‐related	5.23E−07
17.6.1.13	At1g30040	GA2ox2 | Gibberellin 2‐oxidase	4.5E−06
17.6.3	At5g15230	GASA4 | GAST1 protein homolog 4	2.62E−05
17.1.1	At1g16540	ABA3 | ABA deficient 3	1.64E−05

We identified 16 and 15 Arabidopsis accessions carrying the minor frequency allele for *ABA3* and *GA2ox2* candidate genes, respectively, which showed reduced sensitivity to IAM (Fig. 1e,f). Analysis of the geographic distribution of these alleles revealed distinct patterns for both candidate genes. While *GA2ox2* alleles exhibited a dispersed distribution, the *ABA3* alternative allele was confined to a smaller geographic area in southwest Iberia (Fig. 1e,f). Notably, 13 out of the 16 accessions with the minor frequency allele of *ABA3* belong to the 14 accessions containing genetic group C4 previously described in the Iberian Peninsula (Tabas‐Madrid *et al*., 2018), which has been characterized for a relevant adaptive trait dependent on ABA signaling, such as seed dormancy (Vidigal *et al*., 2016).

### Sequence analysis revealed strong linkage disequilibrium and structural polymorphisms in *ABA3*

**Fig. 2:**
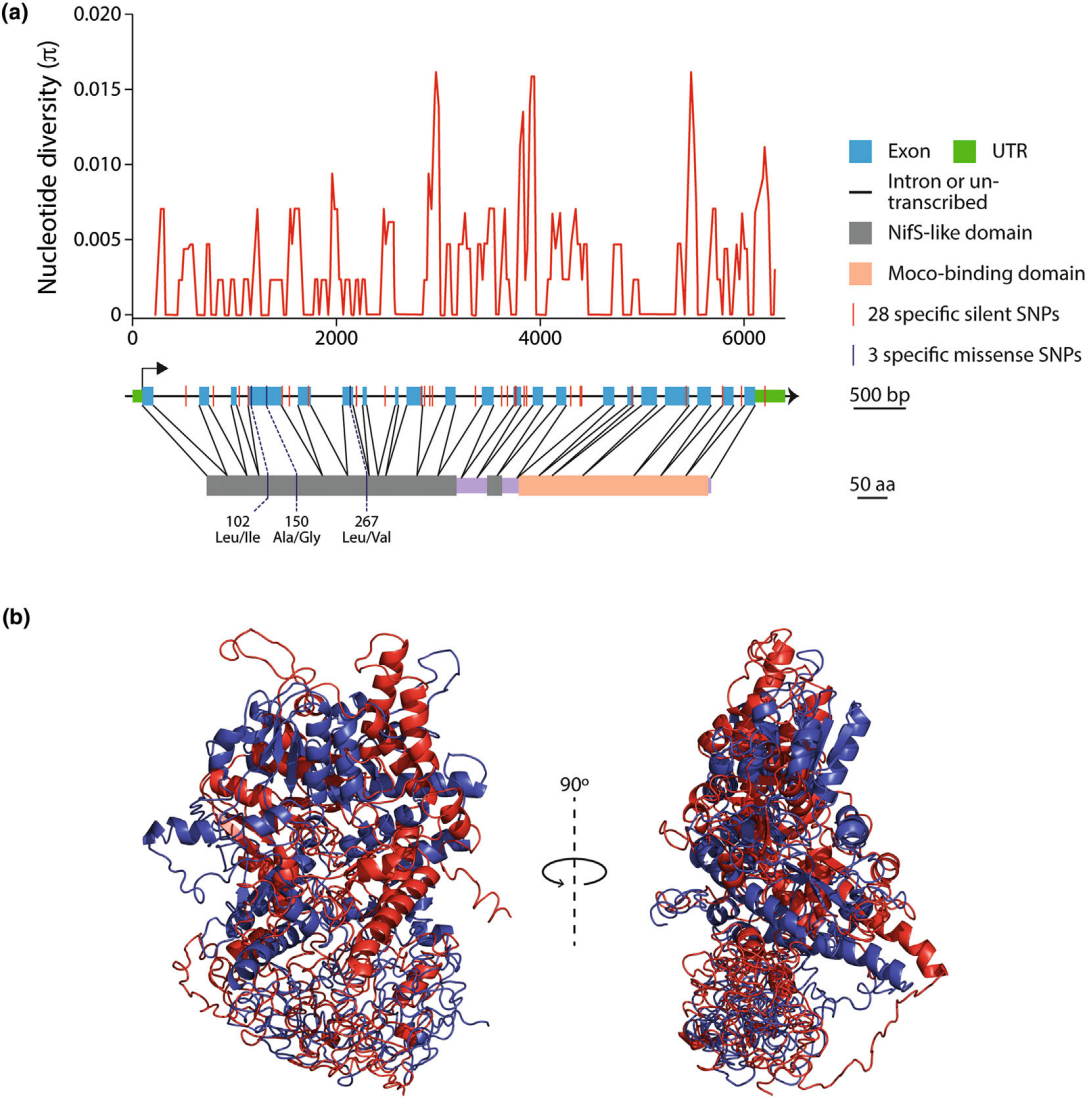
Analysis of the nucleotide diversity of the Arabidopsis *ABA3* gene. (a) Gene organization and nucleotide polymorphisms in *ABA3*. (b) Superimposition of the predicted 3D structure models for ABA3_Ref (blue) and ABA3_Alt (red). The models have been inferred using the I‐TASSER structural prediction server.

To investigate the two selected candidate genes in more detail, we analyzed the quantity and distribution of genetic diversity in their genomic DNA sequences of 166 accessions. A total of 155 (95 in the promoter and 60 in the CDS) and 63 SNPs (37 in the promoter and 6 in the CDS) were segregating for *ABA3* and *GA2ox2*, respectively, corresponding to high and low nucleotide diversities (π‐*ABA3* = 0.00415 (Promoter + CDS); π‐*GA2ox2* = 0.00295 (Promoter + CDS)). As illustrated in Fig. 2, SNPs were distributed throughout the length of the *ABA3* gene and exhibited strong linkage disequilibrium (LD), as the minor alleles were shared by the majority of the 16 Arabidopsis accessions with the minor allele detected by GWAS. Notably, 31 of the polymorphisms were in complete LD in these accessions, defining a highly differentiated allele referred to as ABA3_Alt, which encompassed six synonymous and three missense mutations: L102I, A150G, and L267V. By contrast, consistent with the low nucleotide diversity observed in *GA2ox2*, only two SNPs with partial LD distinguished the alternative allele identified as associated in GWAS analyses, and neither suggested an alteration in the primary amino acid sequence.

In order to assess the influence of missense mutations distinguishing the two alleles identified in *ABA3* that might interfere with the functionality of the translated protein, we conducted an analysis of the secondary and tertiary structures of ABA3 as encoded by both the reference and alternative alleles. Although the three primary amino acid substitutions are conservative exchanges, the prediction of the secondary protein structures indicated minor differences in the length and organization of the α‐helices, β‐strands, and coiled regions (Fig. S1).

Despite the high sequence identity of 99.64%, the reference ABA3 protein from Col‐0 plants exhibits moderate differences from the alternative protein version found in the 16 Arabidopsis accessions. As illustrated in Fig. 2b, the structural comparison of the homology‐based protein models derived for ABA3_Ref and ABA3_Alt, obtained from the I‐TASSER prediction server (Yang & Zhang, 2015) revealed that changes in the secondary structure of the two proteins encompass a slight torsion of ABA3_Alt relative to ABA3_Ref. Such structural modifications can influence the geometry and steric properties of a protein. The activity and substrate affinity of enzymes are largely determined by optimal fixed distances between the amino acids of the catalytic center, as well as between the catalytic center and the cofactor binding sites. In this context, the L267V mutation, in particular, could interfere with the binding of the pyridoxal phosphate cofactor to the conserved K271 residue in the NifS‐like domain of the enzyme, which is essential for ABA3 activity (Heidenreich *et al*., 2005; Schwarz *et al*., 2009). Additionally, the mutations could affect protein stability. Therefore, the potential impact of the three mutations on protein stability was estimated using an integrative computational approach employing the DUET and the I‐Mutant2.0 servers (Capriotti *et al*., 2005; Pires *et al*., 2014). While the ∆∆G values presented in Table 2 showed minor discrepancies between the two tools, they consistently remained negative for all three mutations, indicating a predicted minor decrease in the stability of ABA3_Alt compared to ABA3_Ref.

**Table 2:** Calculation of Gibbs free energy of unfolding (∆*G*) of the modified ABA3 protein (ABA3_alt) variant found in the 16 identified Arabidopsis accessions compared to the reference protein (ABA3_Ref) from Col‐0.

Mutation	∆∆*G*_AltRef_ = ∆*G*_Alt_ – ∆*G*_Ref_	
	DUET (kcal mol^−1^)	I‐Mutant2.0 (kcal mol^−1^)
L102I	−0.24	−0.83
A150G	−0.82	−1.17
L267V	−1.28	−0.94

∆∆*G* < 0, decrease stability; ∆∆*G* > 0, increase stability.

### ABA3 affects the IAM triggered reduction of primary root growth

To further empirically investigate the role of ABA3 and GA2ox2 in the IAM‐mediated growth repression, we obtained the corresponding knockout lines for both genes. The *GA2ox2* gene is a member of a small isogene family of C19‐GA2 oxidases comprising five members, *GA2ox1*, ‐*2*, *‐3*, *‐4*, and ‐*6*, which are reported to inactivate physiologically active GA through its oxidation (Rieu *et al*., 2008). In previous studies, we observed that not only *GA2ox2* but also *GA2ox6* is induced in the *ami1‐2* mutant background that is impaired in the conversion of IAM into IAA (Pérez‐Alonso *et al*., 2021). Furthermore, previous work identified a potential role of GA signaling in the response to IAM accumulation in the *ami1‐2 rty1‐1* double mutant (Sánchez‐Parra *et al*., 2021). Consequently, we opted to incorporate the previously described *ga2ox1 ga2ox2 ga2ox3 ga2ox4 ga2ox6* quintuple mutant (*ga2ox q*) in the study to minimize the potential compensation of the *ga2ox2* mutation by the other isoforms.

ABA3 is a molybdenum cofactor sulfurase that is essential for the activation of aldehyde oxidases, such as ARABIDOPSIS ALDEHYDE OXIDASE 3 (AAO3), which catalyzes the final step in ABA biosynthesis (Seo *et al*., 2000; Bittner *et al*., 2001). For this reason, we decided to investigate the role of ABA biosynthesis and signaling in the processing of the IAM stimulus by analyzing the *aba3‐1* and *abi5‐7* knockout mutants, respectively.

**Fig. 3:**
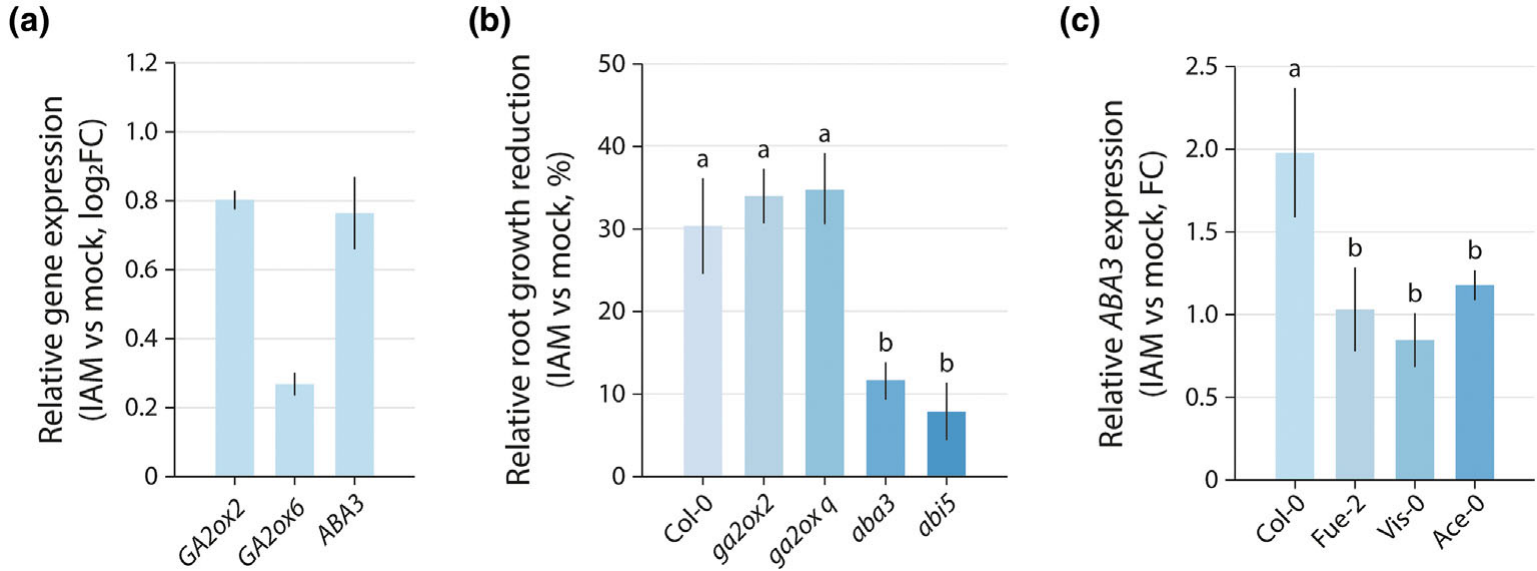
Transcriptomics and reverse genetics analysis of GA degradation and abscisic acid (ABA) biosynthesis‐ and signaling‐related genes. (a) Quantification of *GA2ox2*, *GA2ox6*, and *ABA3* expression in Arabidopsis wild‐type (Col‐0) seedlings. The plot presents means ± SE (*n* = 9) of the relative expression levels comparing the samples from mock‐treated plants with plants that were treated for 2 h with 10 μM IAM. (b) Comparative analysis of indole‐3‐acetamide (IAM)‐dependent relative primary root growth repression (RPRR) in GA degradation (*ga2ox2*, *ga2ox6*), ABA biosynthesis (*aba3*), and ABA signaling (*abi5*) mutants. The values represent the means ± SE (*n* = 24) of the root growth reduction relative to the length of wild‐type (Col‐0) Arabidopsis plants grown under control conditions. (c) IAM‐mediated *ABA3* induction in three IAM insensitive Arabidopsis accessions compared to the Col‐0 reference accession. For the statistical analysis, one‐way ANOVA with a *post hoc* Tukey–Kramer test was employed. Different letters indicate significant differences between means (*P* ≤ 0.05).

First, we confirmed the previously described transcriptional regulation of *GA2ox2*, *GA2ox6*, and *ABA3* by IAM in the Col‐0 reference ecotype (Ortiz‐García *et al*., 2022). The gene expression levels in response to a short‐term treatment with 10 μM IAM relative to mock‐treated plants in wild‐type Arabidopsis were analyzed by qPCR analysis. As demonstrated in Fig. 3a, the three target genes exhibited moderate induction by IAM. The most pronounced upregulation was observed for *GA2ox2*, followed by *ABA3*. The effect of IAM on the expression of *GA2ox6* was minimal.

The observed transcriptional responses indicated an enhancement in the catabolism of bioactive GA concomitant with the upregulation of ABA biosynthesis. Subsequently, we investigated the root growth response in the different IAM‐ and mock‐treated Arabidopsis mutants. As all mutant lines shared the same Col‐0 background, this wild‐type accession was utilized as a reference to evaluate the impact of the different mutations on IAM‐dependent root growth inhibition. As illustrated in Fig. 3b, Col‐0 control seedlings exhibited 30% shorter primary roots when treated with IAM. For the *ga2ox2* and *ga2ox q* mutants, a 34% and 35% reduction of primary root growth in IAM‐treated seedlings was observed, respectively. However, these latter values of root growth reduction were not significantly different from the wild‐type control. By contrast, ABA biosynthesis and signaling mutants demonstrated a significantly lower inhibition of root growth triggered by IAM. For the *aba3* mutant, only a 12% reduction of primary root growth was recorded, whereas the ABA signaling mutant, *abi5*, exhibited only an 8% reduction. It is noteworthy that the *aba3* and *abi5* mutations had a similar significant effect on IAM‐triggered primary root growth inhibition, thus suggesting that ABA synthesis and signaling are equally important for this phenotype. Collectively, these findings indicate that IAM‐induced ABA biosynthesis and signaling events contribute to the IAM‐triggered root growth inhibition observed in wild‐type Col‐0 control plants, while C19‐GA2 oxidases are likely not involved in the generation of the root growth response to IAM. Subsequently, we investigated whether the transcriptional regulation of *ABA3* gene expression differed between the Col‐0 plants and plants from three selected representative IAM‐insensitive Arabidopsis accessions (Fig. 3c) that carry the minority allele for *ABA3* and display a reduced IAM effect on primary root growth (Table S1). qPCR analysis revealed a significant difference in *ABA3* induction in the accessions Fue‐2, Vis‐0, and Ace‐0 compared to Col‐0. In contrast to Col‐0, which demonstrated the anticipated induction of *ABA3* following short‐term treatment with IAM, two of the three selected accessions did not exhibit any significant induction of the gene. Moreover, the induction of *ABA3* observed in Ace‐3 was < 20% of that in Col‐0 and did not significantly differ from the *ABA3* induction levels in Fue‐2 and Vis‐0. This observation indicates that the insensitivity to IAM is largely attributable to modifications in the transcriptional regulation of *ABA3*.

### IAM triggers ABA signaling in Arabidopsis roots

**Fig. 4:**
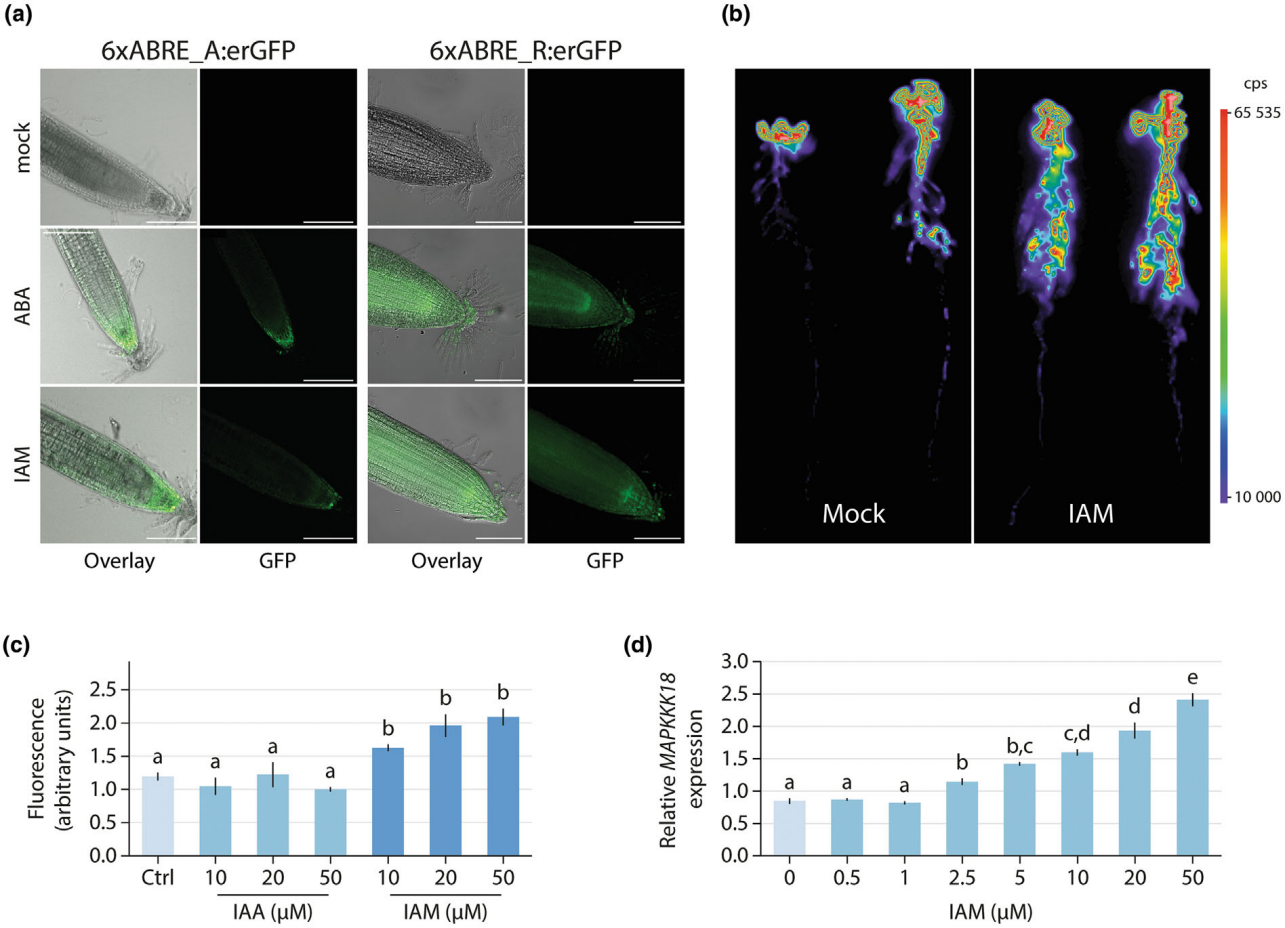
Indole‐3‐acetamide (IAM) induced abscisic acid (ABA) signaling in *Arabidopsis thaliana* plants. (a) Representative images of the effect of a 2 h treatment with 10 μM ABA and 20 μM IAM in comparison to mock controls monitored by using two synthetic promoter reporter lines for ABA signaling. Bars, 100 μm. (b) Confirmation of IAM triggered ABA signaling at the whole plant level using the pMAPKKK18::Luc^+^ bioluminescence promoter reporter line. The color scale gives the bioluminescence value. (c) Quantification of the average fluorescence signal in synthetic ABA reporter lines following mock treatment or the application of increasing concentrations of IAA and IAM, as indicated in the image. The graph illustrates the means ± SE (*n* = 5). (d) The induction of the *MAPKKK18* ABA signaling reporter gene by IAM was quantified using quantitative polymerase chain reaction (qPCR) analysis. Arabidopsis Col‐0 seedlings were initially grown for 10 d on plates with 0.5× MS medium supplemented with 1% sucrose. They were then transferred to 0.5× MS liquid medium containing the increasing concentrations of IAM indicated in the graph. After a 2 h incubation, total RNA was extracted to assess the expression of *MAPKKK18* levels. The bar plot shows the means ± SE (*n* = 9). Statistical significance was determined using one‐way ANOVA with a *post hoc* Tukey–Kramer test. Different letters indicate significant differences between means (*P* ≤ 0.05).

Given the substantial impairment of ABA biosynthesis and signaling mutants in the response to IAM and the previously documented impact of endogenous IAM accumulation on cellular ABA levels (Pérez‐Alonso *et al*., 2021), we postulated that IAM application induces ABA synthesis and subsequent signaling in Arabidopsis roots. To examine this hypothesis, alterations in ABA signaling were assessed in ABA signaling reporter lines following a short‐term treatment with 20 μM IAM. As illustrated in Fig. 4a, the exposure of the reporter lines to IAM elicited an ABA signaling response comparable to that observed in the ABA‐treated control samples.

Furthermore, we investigated the effect of IAM treatment at the whole plant level. To this end, we examined the effect of IAM treatment on ABA signaling using the pMAPKKK18::Luc^+^ reporter line, which confirmed the promotion of ABA signaling (Fig. 4b). The bioluminescence signal was quantified under these conditions, and the induction of the *MAPKKK18* gene by IAM was confirmed through qRT‐PCR analysis (Fig. S2). It is noteworthy that the observed effect was predominantly localized to the roots.

Subsequently, we investigated whether ABA signaling is specifically triggered by IAM or if IAA could also induce ABA signaling in the 6xABRE_A::erGFP and 6xABRE_R::erGFP reporter lines. To address this question, the reporter lines were either mock‐treated or subjected to a 2 h short‐term treatment with IAA and IAM, respectively, at increasing concentrations (Fig. 4c). The quantification of the fluorescence provided evidence for a statistically significant increase in ABA signaling induction when the reporter lines were treated with IAM. Conversely, the IAA treatment had no significant effect on ABA signaling, suggesting an IAM‐specific effect. To further explore the dose–response relationship of Arabidopsis roots to IAM, the expression level of the *MAPKKK18* gene was quantified across a wider range of IAM concentrations using qPCR. As depicted in Fig. 4d, a significant upregulation of *MAPKKK18* expression was observed as early as 2 h after treatment with 2.5 μM IAM in the liquid medium. Given the brief incubation period and the indirect measurement of the IAM‐induced effect, which involves the intermediate formation of ABA before the transcriptional activation of *MAPKKK18*, the observed threshold of IAM‐induced ABA signaling appears to be at a reasonable level. This largely excludes artificial and merely stress‐related responses to IAM.

### IAM and ABA regulate a common transcriptional response module

**Fig. 5:**
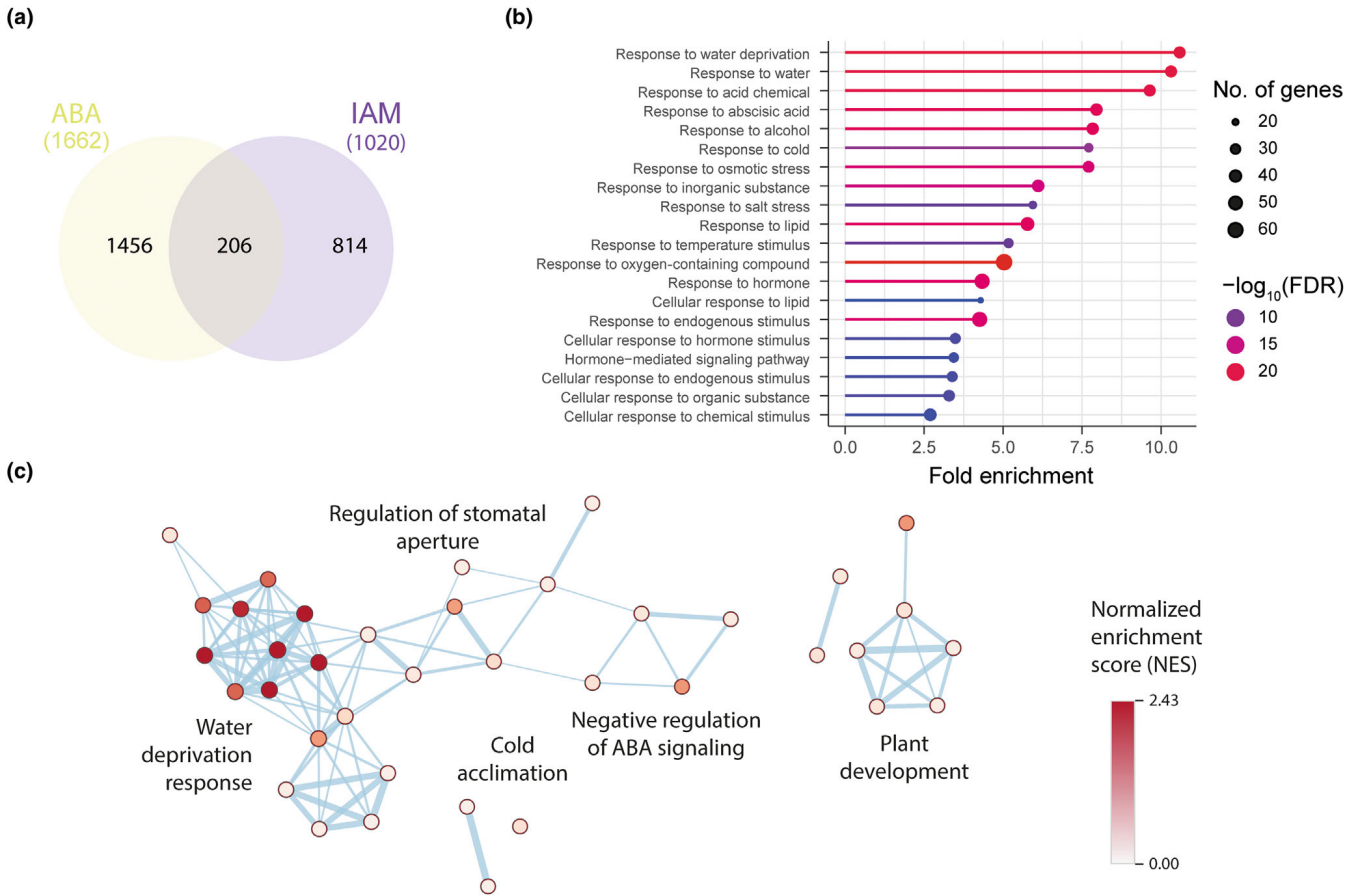
Comparison of indole‐3‐acetamide (IAM) and abscisic acid (ABA) regulated genes in Arabidopsis. (a) Venn diagram analysis showing the numbers of DEGs in response to a 3 h IAM and ABA treatment. (b) Gene ontology (GO) enrichment analysis of the 206 genes in the intersection between IAM and ABA‐regulated differentially expressed genes (DEGs). Each circle in the figure represents a distinct GO term, and the circle size indicates the number of genes enriched in the corresponding GO term. The significance of the observed gene enrichment is represented by a color gradient referring to the −log_10_ (False Discovery Rate (FDR)). (c) GO term enrichment map for the 31 DEGs in the core *RD29B*/*HAI1* network. GO terms that share members are shown in connected clusters. Cluster labels were retrieved using the AutoAnnotate v.1.4.1 application in Cytoscape. The node color intensity reflects the normalized enrichment score (NES).

Considering the likely crosstalk between IAM and ABA, we subsequently examined whether convergent gene regulatory networks could be inferred by comparing existing transcriptomics datasets. To this end, a dataset on IAM‐triggered transcriptional responses in the *ami1* null mutant *ami1‐2* (Ortiz‐García *et al*., 2022) was compared with publicly available data (GSE39384) deposited in the gene expression omnibus (GEO) database (Barrett *et al*., 2012) regarding the response of Arabidopsis seedlings to ABA treatment (Goda *et al*., 2008) (Table S4). As illustrated in Fig. 5, a shared group of 206 DEGs (8.3% of the total compared genes) was identified in the intersection between the 1020 and 1673 DEGs in response to 3 h IAM or ABA treatments, respectively (cutoff of log_2_(fold change) ≥ |1|, FDR ≤ 0.05).

To further characterize the physiological function(s) of genes shared by both ABA and IAM responses, we conducted a gene ontology (GO) analysis and a functional network analysis. A comprehensive analysis of the GO classification of the selected DEGs revealed a potential enrichment of genes in biological processes associated with numerous stress responses (Fig. 5b, Table S4). In addition to an anticipated enrichment of general hormone response‐related GO terms, such as GO:0009725 (response to hormone), GO:0032870 (response to hormone stimulus), and GO:0009755 (hormone‐mediated signaling pathway), and a directly ABA‐related GO term, that is, GO:0009737 (response to ABA), we identified several abiotic stress‐related classifications among the significantly enriched GO terms. The GO classification with the highest enrichment was GO:0009414 (response to water deprivation), followed by GO:0006970 (response to osmotic stress), GO:0009409 (response to cold), and GO:0009651 (response to salt). These GO terms indicate the involvement of the IAM and ABA regulated target genes in the adjustment of the cellular osmotic or solute potential (ΨS).

Subsequently, a functional association network analysis was conducted using the stringApp in Cytoscape for the 206 selected genes, resulting in a network with 206 nodes and 245 edges. Analysis of the network topology identified the genes *RESPONSIVE TO DESICCATION 29B* (*RD29B*) and *HIGHLY ABA INDUCED PP2C GENE 1* (*HAI1*) as central components in the network. *RD29B* exhibited the highest degree of connectivity with 22 connections, while *HAI1* demonstrated the highest betweenness centrality of the core network. Fig. 5c illustrates the subnetwork inferred from *RD29B* and *HAI1* and their 29 directly connected genes, including the PP2C genes *ABA‐INSENSITIVE 1* (*ABI1*), *ABI2*, *HYPERSENSITIVE TO ABA 1* (*HAB1*), and *HAI2*. Moreover, *RD29B* and *HAI1* appeared to be functionally associated with a series of TFs, including *HB‐7* and *HB‐12*, two Homeodomain‐Leucine Zipper subfamily I (HD‐ZIP I) TFs (Ré *et al*., 2014), the NAM, ATAF1/2 and CUC2 (NAC) family TFs *NAC019* (Sukiran *et al*., 2019), *NAC032* (Maki *et al*., 2019), and *NAC092|NAC6|ORE1* (Escamez *et al*., 2020), as well as the MYELOBLASTOSIS (MYB) family TF *MYB74* (Ortiz‐García *et al*., 2022). Subsequently, the 31 genes in the *RD29B/HAI1* subnetwork were subjected to an enrichment analysis using the Enrichment Map application. The nodes in Fig. 5c represent biological processes, and the edges represent gene crosstalk between the different processes (calculated using *P* < 0.05, FDR < 0.1, and the Jaccard coefficient cutoff of 0.25), which links biological processes that share a substantial proportion of genes, thus reducing the redundancy that exists in GO databases. In summary, biological processes related to the response to water deprivation, including adjustment of the cellular osmotic potential and plant growth, are the most prominent physiological functions regulated by the overlapping action of IAM and ABA.

## Discussion

The regulation of root development is highly intricate and essential for plant fitness (Motte *et al*., 2019). The root system anchors the plant in the soil and serves as the organ responsible for the absorption of water and inorganic nutrients (Robe & Barberon, 2023). Regarding the latter, roots function as source tissues from which nutrients are subsequently distributed to sink tissues, including the photosynthetically active aerial plant organs, in a meticulously regulated manner (Chang & Zhu, 2017).

Our previous studies highlighted a growth‐inhibiting effect of IAM on primary roots. Mutations in the *AMI1* gene caused an accumulation of IAM in the seedlings and led to a substantial change in the root phenotype characterized by shorter roots and a reduced root branching density. In addition, IAM treatments of *ami1* knockout and conditional *AMI1* overexpression mutants resulted in longer and shorter primary roots, respectively, which were partly attributed to the conversion of IAM to IAA by IAM‐specific amidases (Pérez‐Alonso *et al*., 2021). By contrast, another study put further emphasis on the growth‐inhibiting effect of IAM on Arabidopsis seedlings. With the objective to rescue mutants in the *SUPERROOT 1*|*ROOTY* gene, the *rty1‐1* mutant was crossed with the *ami1‐2* mutant to block the increased conversion of IAM to IAA in this mutant (Sugawara *et al*., 2009). However, the *ami1‐2 rty1‐1* double mutant showed an unexpected growth arrest resulting in nonviable seedlings, which was traced back to a massive accumulation of IAM in the double mutant (Sánchez‐Parra *et al*., 2021).

In this study, we employed a GWAS approach to advance our understanding of the role of IAM in regulating the development of the root system. By utilizing natural variations as a molecular tool, we aimed to identify novel components associated with IAM‐mediated root growth inhibition. Phenotypic analysis of 166 Arabidopsis accessions from an Iberian Peninsula collection (Arteaga *et al*., 2021) revealed substantial variation in the root response to IAM in the growth medium, which varied between hypersensitive accessions, such as Ria‐0 with a nearly 65% reduction of primary root length, and hyposensitive accessions, such as Ace‐0 that showed no response to IAM or even growth induction relative to the control (Fig. 1). Notably, among the 241 genes with significant associations, only six were found to be directly related to plant hormones (Table 1). We further investigated two of these genes, *ABA3* and *GA2ox2*, as they have been previously shown to play a role in the differential responses to IAM across various genotypes (Ortiz‐García *et al*., 2022). In the case of *GA2ox2*, the most significantly associated SNP 10535569 was found 1888 bps upstream of the gene, which showed no strong LD with SNPs within the coding sequence of the gene. Moreover, the analysis of the individual and quintuple *ga2ox* mutants failed to show any implication of GA2ox proteins in IAM‐mediated root growth inhibition. On the contrary, for the *ABA3* gene, the minor frequency allele at the most significant SNP in position 5659 755 was shared by 16 accessions. This SNP was in complete LD with numerous SNPs in the coding sequence, including three missense mutations, which led us to compare the predicted three‐dimensional structure of the ABA3 protein between the shared alternative variant (ABA3_Alt) and the Col‐0 reference version (ABA3_Ref). This analysis suggested minor structural changes in the ABA3 protein encoded by the specific Iberian allele (Fig. 2). Notably, it was observed that certain representative IAM‐insensitive accessions exhibited only minimal if any transcriptional response to IAM, which is likely to be attributed to the considerably high nucleotide diversity of π‐*ABA3* = 0.0073 detected for the promoter region of the 16 identified accessions (Fig. 3c). Furthermore, accessions with this allele belong to an Iberian‐specific genetic lineage that is distributed exclusively in South‐western Iberia (Fig. 1), a geographic region characterized by high average temperature and low precipitation (Brennan *et al*., 2014). This climatic distribution has been previously shown to likely impact the life‐cycle phenology of Arabidopsis populations belonging to this genetic lineage, as supported by the positive correlation between mean annual temperature and seed dormancy in this region (Vidigal *et al*., 2016; Marcer *et al*., 2018). Therefore, the *ABA3* allele of these accessions might contribute to adaptation to such environmental conditions, although, given the broad genetic differentiation of this lineage, we cannot discard that such adaptation is caused by other correlated genetic components (Picó *et al*., 2008; Brennan *et al*., 2014).

Given the observed insensitivity to IAM in the identified accessions possessing the minor frequency allele of *ABA3*, we initially hypothesized that the resultant ABA3_Alt protein possibly exhibits diminished enzymatic activity, leading to a significant reduction in ABA content. Although the proposed explanation is supported by the predicted reduced stability of the alternative protein, it does not align with the previously documented high seed dormancy, as indicated by DSDS50 (days of seed dry storage required to achieve 50% germination) values > 400 for the identified accessions (Vidigal *et al*., 2016). Seed dormancy is a crucial adaptive trait that optimizes seed germination in response to favorable environmental conditions. The regulation of seed dormancy and germination is predominantly governed by the intricate interaction between GA and ABA biosynthesis and signaling pathways (Liu & Hou, 2018). It is widely recognized that ABA serves as a significant positive regulator of seed dormancy (Finch‐Savage & Leubner‐Metzger, 2006). A deficiency in ABA during seed development leads to a lack of primary dormancy, whereas excessive ABA production during seed maturation enhances dormancy and typically results in delayed germination (Nambara & Marion‐Poll, 2003). Consequently, a general reduction in ABA synthesis through less active versions of the enzyme in accessions possessing the minor frequency allele of *ABA3* must be ruled out. On the contrary, the reduced or absent transcriptional response to IAM in the tested IAM‐insensitive accessions appears to be the more reasonable cause for the observed IAM insensitivity. Consequently, it must be concluded that alterations in the transcriptional regulation of the *ABA3* gene by IAM are responsible for the abnormal root growth phenotype in accessions outside of the ones that were identified to carry the minor frequency allele for *ABA3*. Given the distribution of the identified accessions (Fig. 1e) and the previously mentioned climatic conditions in this region, along with the temperature‐dependent regulation of *AMI1* gene expression (Fig. S3), which is repressed only by cold and not by heat (Waese *et al*., 2017), it can be hypothesized that the identified IAM‐insensitive accessions have lost or never developed the ability to respond to increased IAM levels due to the repression of *AMI1* expression, as they rarely experience cold stress in their natural environment.

Given the significant reduction in IAM‐dependent inhibition of primary root growth observed in the *aba3* ABA biosynthesis mutant and the *abi5* ABA signaling mutant (Fig. 3b), we deduce that IAM‐induced ABA synthesis, facilitated by the induction of *ABA3* (Fig. 3a), is crucial for the repression of root growth in the reference Arabidopsis accession Col‐0. The functional association between ABA3 and IAM‐mediated root growth repression provides a link that integrates the IAM pathway of auxin biosynthesis with the synthesis of the stress hormone ABA. To empirically establish a connection between IAM and subsequent ABA signaling events, we hypothesized that IAM treatment should induce discernible changes in ABA signaling. In support of this hypothesis, the treatment of ABA signaling reporter lines with IAM demonstrated a substantial activation of ABA signaling and indicated that the effect of IAM was primarily confined to the roots (Fig. 4a,b). Crucially, it was observed that increasing levels of IAM significantly induced ABA signaling, whereas similar experiments with IAA did not lead to a notable induction of ABA signaling. These results imply that IAM may have a function beyond its role as an intermediate in a secondary pathway of auxin biosynthesis, potentially acting as an independent signaling molecule (Fig. 4c). Furthermore, we conducted a quantitative assessment of the dose dependency of IAM‐induced activation of the ABA reporter gene *MAPKKK18* in Arabidopsis roots using qPCR analysis (Fig. 4d). Notably, at a concentration of 2.5 μM IAM in the medium and following a brief incubation period of only 2 h, we observed a significant induction of *MAPKKK18*, largely ruling out general stress‐related phenomena.

The physiological and molecular functions of ABA as a signaling molecule in plant responses to abiotic stressors are well established. An increase in cellular ABA levels has been convincingly linked to salt (Jia *et al*., 2002; Holsteens *et al*., 2022), cold (Shinkawa *et al*., 2013), and drought stress (Kuromori *et al*., 2018). At low concentrations (0.1 μM), ABA promotes root growth in a dose‐dependent manner, whereas treatments with 1 μM ABA inhibit root growth (Ghassemian *et al*., 2000; Yoshida *et al*., 2019). The inhibitory effect of high ABA concentrations on root growth involves the suppression of cell division in the apical meristems and the repression of cell expansion in the root elongation zone (Takatsuka & Umeda, 2014; Yang *et al*., 2014). Moreover, the growth inhibitory effects are further mediated by components of the ABA signaling cascade. Several of the 14 ABA receptor molecules in Arabidopsis, such as PYR1, PYL1, PYL2, PYL4, PYL5, and PYL8, act redundantly, thus contributing together to the inhibition of primary root growth (Park *et al*., 2009; Gonzalez‐Guzman *et al*., 2012; Antoni *et al*., 2013). After ABA binding to the receptors, the negative coregulators ABI1, ABI2, HAB1, and the PP2CAs are sequestered by the receptors and thereby inactivated (Rubio *et al*., 2009; Thole *et al*., 2014). This liberates the sucrose nonfermenting 1 related protein kinases (SnRKs) SnRK2.2, SnRK2.3, and SnRK2.6 that act downstream of the PP2Cs and promote the ABA inhibition of primary root growth (Fujii *et al*., 2007; Zheng *et al*., 2010). The comparative transcriptomics analysis of DEGs between the IAM accumulating *ami1‐2* mutant and ABA treatment underpinned a substantial overlap of the responses. More than 20% of the DEGs in the *ami1‐2* mutant were also found to respond to ABA. The classification of the 206 DEGs in the intersection pointed towards an involvement in responses to drought, cold, and osmotic stress (Fig. 5). A functional network analysis revealed a core subnetwork comprising a considerable number of PP2C type protein phosphatases, including *ABI1*, *ABI2*, *HAB1*, as well as *HAI1* and *HAI2*. The induction of these negative regulatory components possibly points to an involvement of IAM in the transcriptional control of a feedback loop that negatively regulates drought stress responses, such as the control of osmoregulatory solute accumulation, which involves the clade A PP2Cs HAI1, HAI2, and HAI3 (Bhaskara *et al*., 2012).

**Fig. 6:**
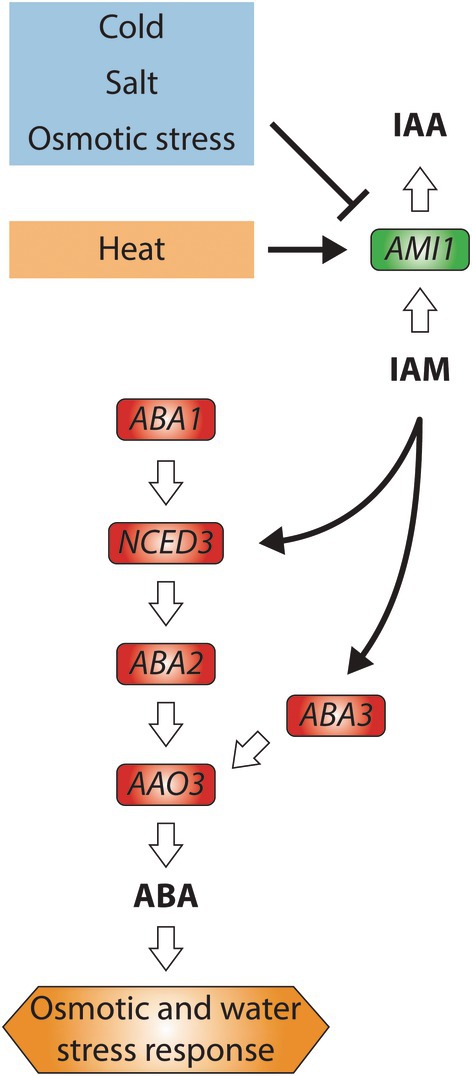
Proposed model for the regulation of abscisic acid (ABA) biosynthesis by indole‐3‐acetamide (IAM). Straight white arrows denote enzymatic processes, whereas black lines refer to regulatory processes. Solid arrows refer to gene inductions, while blunt‐ended arrows indicate a repression of gene expression. Bold letters emphasize signaling molecules. Abiotic stress conditions regulate the expression of *AMI1*, consequently influencing the levels of IAM. The accumulation of IAM enhances the expression of ABA biosynthetic genes, including *NCED3* and *ABA3*. The upregulation of these genes results in elevated ABA levels and signaling, which subsequently govern downstream processes associated with osmotic and water stress responses.

In summary, our research presents novel evidence supporting the involvement of *ABA3* in IAM‐mediated primary root growth inhibition. Consistent with the previously documented induction of *NCED3* and the resultant elevated ABA levels in the *ami1*‐mutant alleles (Pérez‐Alonso *et al*., 2021), the observed induction of *ABA3* further substantiates the proposed model illustrated in Fig. 6. According to data extractable from the ePlant webtool (Waese *et al*., 2017), abiotic stress signals, such as cold, salt, and osmotic stress, exert a negative regulatory effect on the expression of *AMI1* (Fig. S3). This regulation leads to increased levels of IAM, which in turn enhance ABA biosynthesis through the induction of *ABA3*, thereby attributing a signaling molecule role to IAM. Consequently, when *AMI1* expression is diminished in response to abiotic stimuli, an elevation in *ABA3* expression can be anticipated; a hypothesis that is validated by transcriptomic data accessible in public repositories (Fig. S3). Furthermore, our findings reveal a previously unrecognized shared stress response module that is likely implicated in the regulation of abiotic stress responses in Arabidopsis. Investigating the fundamental mechanism of this identified stress response module, which is proposed to integrate varying levels of IAM, represents an intriguing prospect for future research. The examination of potential target genes for a putative IAM receptor, especially those encoding F‐box proteins and TFs, respectively, among the IAM‐responsive DEGs in both wild‐type and *ami1‐2* mutant plants has yielded inconsistent results (Table S4). While there are no F‐box candidate genes induced by IAM treatments in both wild‐type and *ami1‐2* seedlings, there are only four TFs, that is, *MYB47*, *MYB74*, *MYB102*, and *NGA3*, that showed a robust induction in both genotypes. While *MYB47* and *NGA3* are hardly expressed in roots (Trigueros *et al*., 2009; Cao *et al*., 2025), the two other MYB TFs have previously been studied and appear to be at least partially downstream of ABA. More important, however, is the observation that *ABA3* expression is not altered in those MYB mutants (Ortiz‐García *et al*., 2022). Thus, a primary objective will be to investigate the existence of an IAM receptor and to identify the IAM‐responsive TF that presumably governs the transcriptional response of *ABA3* in Col‐0 but not in the accessions carrying the minor frequency allele of *ABA3*.

## Competing interests

None declared.

## Author contributions

SP, JV‐C, JP‐A, CA‐B conceived and designed the research; JM‐C, PO‐G, AGO‐V, IV‐L, AP‐G, JV‐C, and SP performed the research and analyzed the data; SP, JV‐C, JP‐A, and CA‐B were responsible for the acquisition of the required funding to perform the experiments and wrote and edited the manuscript. JM‐C and PO‐G contributed equally to this work. All authors have read and agreed to the published version of the manuscript.

## Disclaimer

The New Phytologist Foundation remains neutral with regard to jurisdictional claims in maps and in any institutional affiliations.

## Supporting information


**Fig. S1.** Secondary structure comparison of ABA3_Alt and ABA3_Ref.
**Fig. S2** Quantification of changes in *MAPKKK18* gene expression in response to IAM treatments.
**Fig. S3** Transcriptional regulation of *AMI1* and *ABA3* by abiotic stress stimuli.

**Table S1** Metadata of the employed *Arabidopsis thaliana* accessions.

**Table S2** Primers used in this study.

**Table S3** List of candidate genes obtained in the GWA study.

**Table S4** Transcriptional response to ABA and IAM treatments and Gene ontology (GO) enrichment analyses.
Please note: Wiley is not responsible for the content or functionality of any Supporting Information supplied by the authors. Any queries (other than missing material) should be directed to the *New Phytologist* Central Office.

## Data Availability

All data supporting the conclusions of this study are present in the paper and the Supporting Information (Figs S1–S3 and Tables [Link], [Link], [Link], [Link]).
