# Supplementary material for: Identification of a novel link connecting indole‐3‐acetamide with abscisic acid biosynthesis and signaling
Source: New Phytol. 2025 Dec 8;249(5):2452–66. doi: 10.1111/nph.70819 (PMC12873522; doi:10.1111/nph.70819)
Supplement: Supplementary file 1 — Fig. S1. Secondary structure comparison of ABA3_Alt and ABA3_Ref. Fig. S2 Quantification of changes in MAPKKK18 gene expression in response to IAM treatments. Fig. S3 Transcriptional regulation of AMI1 and ABA3 by abiotic stress stimuli. [file NPH-249-2452-s004.docx]

## *New Phytologist* Supporting Information

**Article title:** Identification of a novel link connecting indole-3-acetamide with abscisic acid biosynthesis and signaling

**Authors:** José Moya-Cuevas, Paloma Ortiz-García, Adrián González Ortega-Villaizán, Irene Viguera-Leza, Andrés Pérez-González, Javier Paz-Ares, Carlos Alonso-Blanco, Jesús Vicente-Carbajosa, Stephan Pollmann

**Article acceptance date:** Click here to enter a date.

The following Supporting Information is available for this article:

**Fig. S1** Secondary structure comparison of ABA3_Alt and ABA3_Ref

**Fig. S2** Quantification of changes in *MAPKKK18* gene expression in response to IAM treatments

**Fig. S3** Transcriptional regulation of *AMI1* and *ABA3* by abiotic stress stimuli

**Table S1** Metadata of the employed *Arabidopsis thaliana* accessions

**Table S2** List of candidate genes obtained in the GWA study

**Table S3** Transcriptional response to ABA and IAM treatments and Gene ontology (GO) enrichment analysis

**Table S4** Primers used in this study

**Fig. S1** Comparative protein secondary structure prediction for ABA3_Alt and ABA3_Ref using the PSIPRED tool (McGuffin *et al.*, 2000). The color coding for helices, β-sheets, and coiled regions is given in the included legend.

**
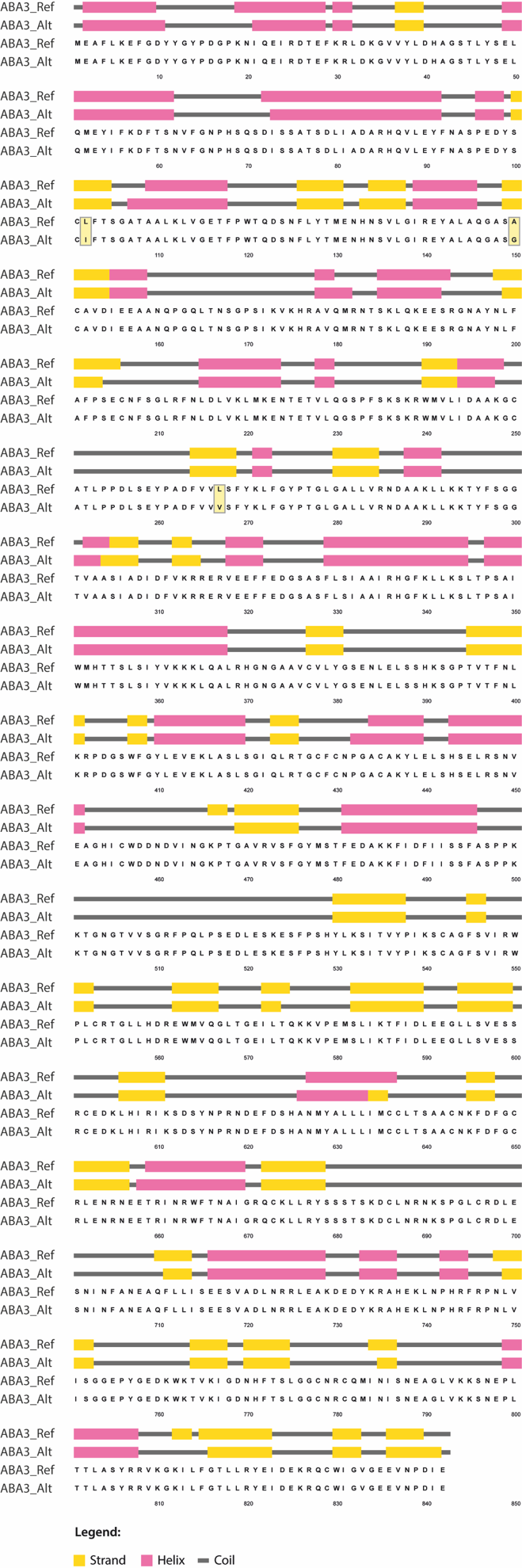
**

**Fig. S2** Quantification of changes in MAPKKK18 gene expression in response to IAM treatments (a) Quantification of the average bioluminescence signal in mock and IAM treated pMAPKKK18::Luc+ ABA signaling reporter lines. The graph depicts means ± SE (n = 5). Student’s t-test: ***p < 0.001. (b) Quantification of transcriptional alterations of the MAPKKK18 (At1g05100) gene in response to a 2 h treatment with 20 μM IAM by qPCR.


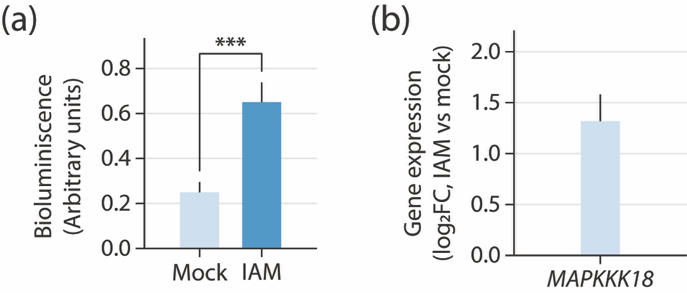


**Fig. S3** Transcriptional regulation of AMI1 gene expression by abiotic stress cues extracted from the ePlant webtool (Waese et al., 2017). (a) The transcriptomics data highlight a considerable suppression of the AMI1 gene by osmotic, salt, and cold stress, while heat appears to induce the expression of the gene. (b) Illustration of gene expression levels for AMI1 and ABA3 across all abiotic stress-related data sets in the ePlant database. The color-coded gene expression levels (from red = high expression to yellow = low expression) largely confirm that ABA3 expression is induced when AMI1 expression is low.

**
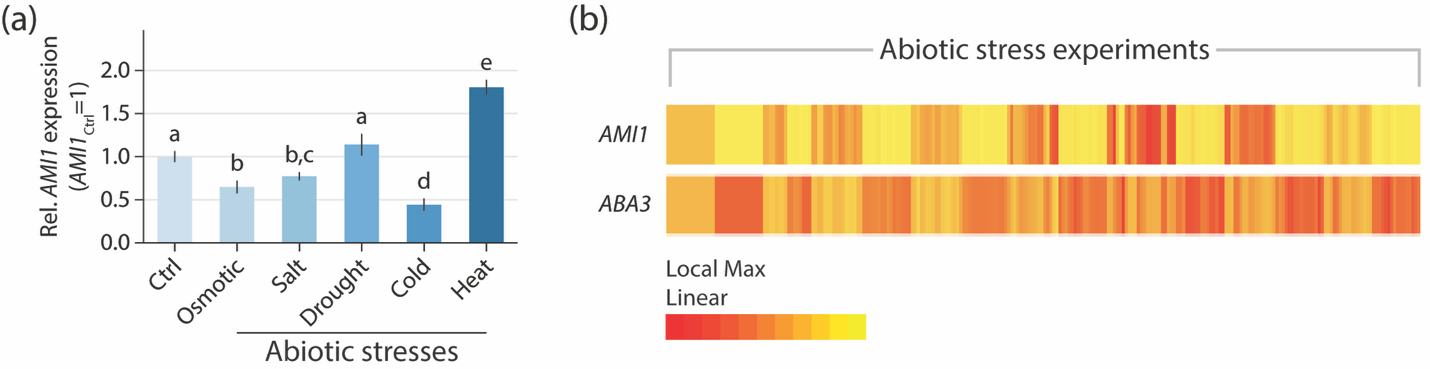
**

**McGuffin LJ, Bryson K, Jones DT. 2000.** The PSIPRED protein structure prediction server. *Bioinformatics* **16**(4): 404–405.

**Waese J, Fan J, Pasha A, Yu H, Fucile G, Shi R, Cumming M, Kelley LA, Sternberg MJ, Krishnakumar V, et al. 2017.** ePlant: Visualizing and Exploring Multiple Levels of Data for Hypothesis Generation in Plant Biology. *Plant Cell* **29**(8): 1806–1821.
